# Critical assessment of the current indicator for antenatal iron‐containing supplementation coverage: Insights from a mixed‐methods study

**DOI:** 10.1111/mcn.13314

**Published:** 2022-01-28

**Authors:** Aatekah Owais, Sara Wuehler, Rebecca Heidkamp, Vrinda Mehra, Lynnette M. Neufeld, Lisa M. Rogers, Kuntal Kumar Saha

**Affiliations:** ^1^ Nutrition International Ottawa Canada; ^2^ Centre for Global Child Health, Research Institute Hospital for Sick Children Toronto Canada; ^3^ International Health, Bloomberg School of Public Health Johns Hopkins University Baltimore Maryland USA; ^4^ Data and Analytics Section, Division of Data, Analysis, Planning and Monitoring UNICEF New York City New York USA; ^5^ Global Alliance for Improved Nutrition (GAIN) Geneva Switzerland; ^6^ Department of Nutrition and Food Safety World Health Organization Geneva Switzerland

**Keywords:** coverage indicator, feasibility, iron‐containing supplements, pregnant women, validity

## Abstract

Daily consumption of iron‐containing supplements is recommended for all pregnant women but there is no approved global standard indicator for assessing supplementation coverage. Furthermore, the validity of commonly used coverage indicators for iron‐containing supplement consumption is questionable. The WHO–UNICEF Technical Expert Advisory Group on Nutrition Monitoring, and partners, have systematically worked to identify a feasible and valid indicator of iron‐containing supplement coverage for reporting by countries. In 2019, we conducted key informant interviews with respondents in eight countries, fielded an online survey (in three languages using SurveyMonkey) to which 142 nutrition professionals from 52 countries responded, and used Demographic and Health Surveys (DHS) data from four countries to assess determinants of the quality of iron‐containing supplement coverage data. Less than half (45%) of online survey respondents were satisfied with the current methods for collecting iron‐containing supplement coverage data in their context. Recommended changes by study respondents include recall period <5 years, adding questions about counselling, including other beneficiary groups, and assessing supply chain functionality. The DHS analysis suggested an association between time since pregnancy and data quality. Data heaping on multiples of 30 was observed in 40%–75% of data. There is a clear demand for a revised indicator and measurement guidance for coverage of iron‐containing supplementation during pregnancy. Future research should continue the development and validation of a global indicator, to more precisely validate the quality of recall data, including the distinction between distribution and consumption using various question formulations.

## Key messages

- Health and nutrition professionals have described concerns with current methods for reporting coverage of daily iron‐containing supplementation in pregnant women.
- The long recall period (5 years) of commonly used iron‐containing supplement consumption indicators calls into question the validity of these indicators.
- Our study indicates that there is demand for a revised indicator and guidance for data collection.

## Graphical abstract

DAG representing hypothesized relationship between iron‐containing supplement consumption recall, maternal sociodemographic factors and recall period.

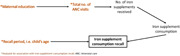

## 1 INTRODUCTION

Anaemia affects an estimated 1.76 billon peoples globally, including 520 million (∼27%) women of reproductive age (GBD 2019 Diseases and Injuries Collaborators, 2020). For women of reproductive age, anaemia, especially iron‐deficiency anaemia, is associated with an increased risk of morbidity and mortality, particularly during pregnancy (Haider et al., 2013; Keats et al., 2021; Rahman et al., 2016; Young et al., 2019). WHO recommends daily supplementation with 30–60 mg elemental iron during pregnancy to prevent adverse maternal and foetal outcomes (WHO, 2016).

Several antenatal iron‐containing supplementation indicators are currently used for reporting in low‐ and middle‐income countries, mostly based on data collected in the Demographic and Health Survey (DHS) women's questionnaire (DHS, 2018). These include various indicators along the coverage continuum including service delivery (e.g., any iron received or purchased), and reported compliance by women during pregnancy (e.g., consumed any, 90, 180 tablets, or other cutoffs). Concerns have been raised about the validity of recall for these indicators because women are reporting on pregnancies completed as long as 5 years preceding the surveys (Kanyangarara et al., 2019). Other relevant data sources for monitoring and reporting on antenatal iron‐containing supplementation include administrative data systems such as Health Management Information Systems (MEASURE‐Evaluation, 2017). These systems generally do not follow individual women over time and are limited to supplement distribution rather than consumption. Furthermore, administrative data have known quality issues (Maiga et al., 2019).

WHO–UNICEF's Technical Expert Advisory Group on Nutrition Monitoring (TEAM) is working towards proposing a global standard indicator on antenatal iron‐containing supplementation coverage. The primary aim is to harmonise reporting of progress in antenatal iron‐containing supplementation programmes by the WHO member states towards the world health assembly 2025 global nutritional target on anaemia. A 2017–2018 feasibility study conducted in eight countries, purposively selected for variability in economic, geographic, and anaemia burden profiles revealed that none of the countries could provide complete information for the indicator proposed by the WHO global nutrition monitoring framework (GNMF) (WHO, 2015, 2018a). TEAM proposed a simplified interim indicator in the 2017 GNMF operational guidance (WHO, 2017) with plans to revisit in the future.

Respondents in TEAM's feasibility study (WHO, 2018a) recommended changes to DHS and similar global survey questionnaires about coverage of iron‐containing supplements during pregnancy, including adding multiple micronutrient supplements, identifying the source of supplements, and whether supplements were provided free of charge.

Our study builds on these efforts to advance the development and validation of a new indicator. We aimed to: 1) identify how iron‐containing supplement consumption data are collected, reported, and used in large‐scale nutrition datasets; and 2) use existing DHS datasets to identify differences in the consistency, quality, and validity of antenatal iron‐containing supplement consumption data.

## 2 METHODS

In early 2019, we carried out a series of key informant interviews (KII) among key stakeholders with experience in a variety of national contexts; feedback and opinions requested related to how iron‐containing supplement consumption data are collected, reported, and used. An online survey was also carried out to obtain similar information from a wider sample of data users, though with less depth possible. In addition, we analysed DHS survey data on coverage of iron‐containing supplements from four countries—Afghanistan (2015), Colombia (2015), Myanmar (2015–2016), and Tanzania (2015–2016) to identify potential determinants of data quality and how these questions might be modified to produce more accurate reports of antenatal iron‐containing supplementation.

### 2.1 Data collection and sources

#### 2.1.1 Key informant interviews

KII covered similar topics and themes as the WHO–UNICEF TEAM feasibility study (WHO, 2018a), but differed in terms of selection criteria. Countries considered for KIIs were identified based on anaemia prevalence among women of reproductive age (15–49 years), as reported in WHO's micronutrients database, as part of the vitamin and mineral nutrition information system (WHO, 2018b) and modelled estimates of the prevalence of anaemia reported by Stevens et al. (2013). Countries with appropriate data (*n* = 79) were categorised into high (prevalence >40%: 30 countries), medium (prevalence 20%–39.9%: 31 countries), and low (prevalence <20%: 18 countries) anaemia burden, as per the thresholds used by WHO for defining the public health significance of anaemia and guiding population‐based public health programmes for iron‐containing supplementation during pregnancy. We randomly selected four countries from each of the three anaemia burden categories (*n* = 12). Potential respondents from each country were purposively selected based on their experience and position within national nutrition programmes and/or country‐specific ministries of health. Contact information for representatives from each country responsible for maternal iron‐containing supplementation programmes and/or related data collection was obtained from or facilitated by the study authors through local contacts or online searches. Individuals were contacted by email or phone with invitations to participate in KIIs. An interview guide for key informants was developed in consultation with TEAM's iron supplement working group (Supporting Information Table A1).

#### 2.1.2 Online surveys

The questions developed for KIIs were also shared as an online survey through the WHO Nutrition Listserv. The survey was open for responses from 25 March to 22 April 2019 and responses were completed by those interested in responding, with no randomisation or representativeness. The questions were available in English, French, and Spanish. The online interface was created using SurveyMonkey (www.surveymonkey.com). Separate survey links were created for each of the three languages.

#### 2.1.3 Demographic and Health Surveys

**Figure 1:**
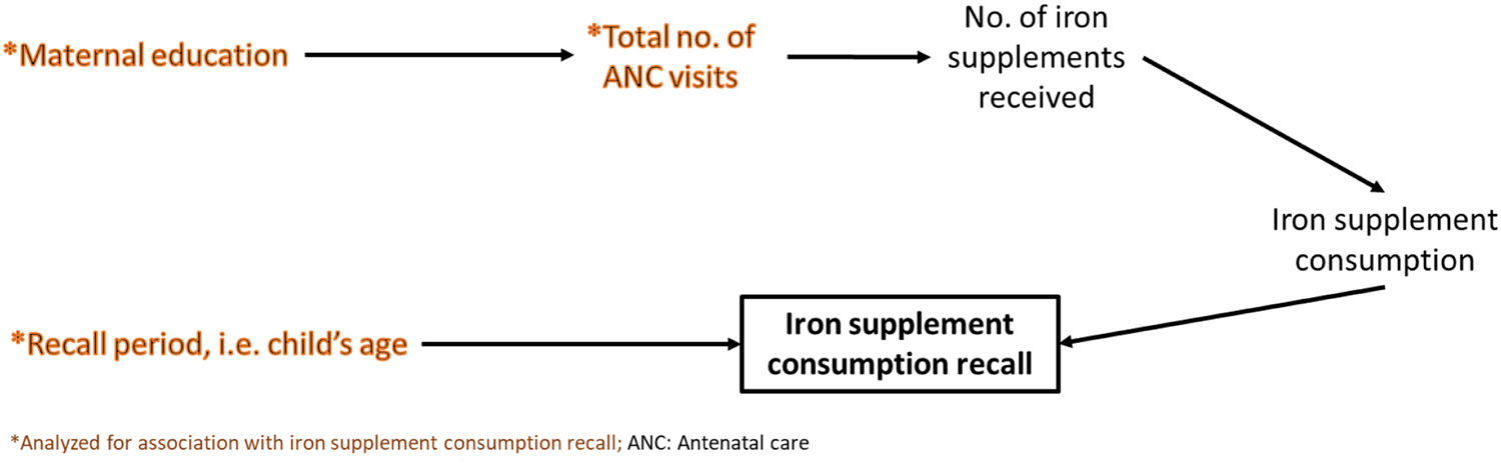
Directed acyclic graph representing the hypothesised relationship between antenatal iron‐containing supplement consumption recall (outcome) and maternal sociodemographic factors and recall period

Countries for DHS data analysis were selected based on the availability of recent data on maternal consumption of iron‐containing supplements (survey conducted post‐2014). Four countries were selected, with two from high anaemia burden countries (Myanmar and Tanzania), one from middle burden countries (Colombia), and one from low anaemia burden countries (Afghanistan). Variables were selected based on known association with maternal consumption of iron‐containing supplements and/or accuracy of recall. For example, maternal education and nutrition knowledge were selected based on research associating these with higher consumption of micronutrient supplements (Nguyen et al., 2019, 2017; Nisar et al., 2014); women who attend more antenatal care (ANC) visits are likely to consume more iron‐containing supplements (Gopalakrishnan et al., 2019; Nguyen et al., 2017; Nisar et al., 2014; Wendt et al., 2015); maternal recall of events which occurred in the prenatal period is associated with maternal education (Stuart et al., 2013); and maternal recall of iron‐containing supplementation receipt has been found to alter after 1–2 years of giving birth (Kanyangarara et al., 2019). In our analysis, we included duration of recall (time between most recent birth and date of survey), reported number of ANC visits, and maternal education as potential determinants of reported consumption of iron‐containing supplements (Figure 1).

### 2.2 Data analysis

#### 2.2.1 Key informant interviews

Although no thematic framework was used to analyse the data collected, questions included in the interview guide focused on the following themes: how were data on antenatal iron‐containing supplementation collected; how were these data used; availability of national supplementation guidelines; availability and distribution mechanism for iron‐containing supplements. Results from KII were tabulated manually around these general themes and summaries are presented narratively.

#### 2.2.2 Online surveys

Responses to the online survey were downloaded and imported into statistical analysis software version 9.4 for analysis. Frequencies and percentages were calculated for all questions. Responses are reported for all individuals who completed the survey.

#### 2.2.3 Demographic and Health Surveys

For women who had a live birth in the 5 years preceding the survey, irrespective of whether they had received ANC, the DHS collects data about whether iron‐containing supplements were ever given or purchased and the number of days/months iron tablets/syrup were consumed. The Afghanistan, Myanmar, and Tanzania surveys collected iron‐containing supplement consumption data in days, whereas Colombia collected data in terms of months.

We restricted our analyses to women who reported consuming iron tablets/syrup for at least 1 day. All analyses of data quality were conducted for each of the four countries separately and adjusted for complex survey design, using country‐specific DHS‐assigned weights. Statistical significance was set at *p* < 0.05.

*Data heaping*: We assessed data heaping in the countries that reported the number of days of antenatal iron tablet/syrup consumed for all women interviewed in the last 5 years as well as by subgroup based on time since birth (<1 year, 1 to <2 years, 2 to <3 years, 3 to <4 years, and 4 to <5 years). We expected to observe a certain amount of heaping at specific values because iron is distributed in packs of distinct quantities (e.g., 30 doses), but anticipated that heaping due to recall bias would increase over time. Three of the four countries included in our study (Afghanistan, Myanmar, and Tanzania) reported intake in days, while one (Colombia) reported intake in terms of months without decimal place (i.e., only full months reported). Therefore, to avoid falsely inflating data reported in multitudes of 30, we did not include Colombia in these analyses. The mean days/months of iron‐containing supplement consumption for each recall period category were compared with the overall mean (i.e., for the cumulative 5 years). We also assessed data heaping descriptively, by comparing the proportion of women who reported multiples of 30 days (i.e., 30, 60, 90, 120, 150, and 180) to those who reported other numbers of supplements consumed.

*Linear regression*: We used linear regression to assess the bivariate relationship between the outcome of number of days (or months) of reported iron‐containing supplement (tablets/syrup) consumption and: (i) time since last completed pregnancy (age of most recent child born to the woman), (ii) number of ANC visits, and (iii) mother's educational attainment (Figure 1). Since women's educational attainment is a strong mediator of ANC utilisation (Ekholuenetale, Benebo, et al., 2020; Ekholuenetale, Nzoputam, et al., 2020; Ousman et al., 2019; Sui et al., 2021), we also assessed the association between educational attainment and iron‐containing supplement consumption, while controlling for number of ANC visits.

*Data quality of iron‐containing supplement consumption data*: The quality of the DHS survey data was assessed using standard error of the mean (SE) and coefficient of variation (CV) calculated around the mean number of days/months during which iron tablets/syrup were consumed, categories of recall period (see above), the total number of ANC visits, and mother's educational attainment (no education, incomplete primary, complete primary, incomplete secondary, complete secondary, higher).

SE estimates how much the sample mean deviates from the true population mean. CV describes the dispersion of the variable of interest (i.e., mean days/months iron tablets/syrup were consumed) and is calculated using the formula: $CV=\frac{SE}{\mu }$, where µ is the sample mean. Higher CV values indicate greater dispersion of the variable, indicating lower reliability of the data, but CVs cannot measure systematic bias. CV values were interpreted as per statistics Canada guidelines (Canadian Travel Survey Microdata User's Guide, 1997) (Supporting Information Table A2).

## 3 RESULTS

### 3.1 Key informant interviews

We completed nine interviews with key informants from eight countries who responded to our request for participation: Afghanistan, Colombia, Guatemala, Japan, Myanmar, Niger, Rwanda, and Tanzania. We replaced five of the original 12 countries randomly selected due to nonresponse by the identified informants yet were still not able to reach at least 12 countries. Interviews were completed between January and March 2019, with each interview lasting 30–40 min. Six key informants worked for a government ministry and three worked for WHO country offices (Supporting Information Table A3).

#### 3.1.1 National guideline for iron‐containing supplement provision to adolescent girls, pregnant and/or lactating women

Key informants from six of the eight countries indicated that a national guideline is currently available for iron‐containing supplement provision to adolescent girls, pregnant and/or lactating women (Supporting Information Table A4). No national guideline exists for Japan; however, the Japan Society of Obstetrics and Gynaecology has a guideline for iron and folic acid supplementation for pregnant women. A national guideline was under development in Rwanda at the time of the research.

#### 3.1.2 Availability of iron‐containing supplements at health facilities

In six of the eight countries, iron‐containing supplements were available free of charge at health facilities where women receive ANC (i.e., first point of contact between a pregnant woman and healthcare provider). In Japan, pregnant women receive a prescription from a healthcare provider to redeem at a pharmacy where they may pay 20%–30% of the cost, depending on their age and income level. In Niger, pregnant women receive iron‐containing supplements free of charge at health clinics with a prescription, or at a charge from pharmacies without a prescription.

#### 3.1.3 Collection of data on coverage/consumption of iron‐containing supplements

A nationally representative household survey with data on iron‐containing supplements was carried out in each of the eight countries within the last 5 years (Supporting Information Table A5). In seven countries, the surveys included coverage or receipt and/or consumption of iron‐containing supplements among pregnant women. The exception was Japan, which measures supplement consumption on the day of the survey for all female survey participants aged 20 years and older, irrespective of pregnancy status. In Colombia and Guatemala, iron‐containing supplement coverage is also assessed using health management information system, which tracks whether women are prescribed iron‐containing supplements during each ANC visit.

Key informants from four of the eight countries indicated that they were not satisfied with how iron‐containing supplement coverage is assessed in national surveys. Reasons included the long recall period (up to 5 years) and the inability to determine actual consumption of iron‐containing supplements by pregnant women. Only the informant from Afghanistan expressed satisfaction with the way iron‐containing supplementation coverage/consumption was currently assessed in national surveys. In Afghanistan, the three most recent surveys that included an assessment of iron‐containing supplementation in pregnant women were the National Nutrition Survey in 2013 (UNICEF, 2013), and Afghanistan Health Survey in 2015 (KIT, 2016) and 2018 (KIT, 2019), and the questions used to ascertain iron‐containing consumption during pregnancy in these surveys were similar to those used in the DHS 2015 (CSO MoPH & ICF, 2017).

#### 3.1.4 Use of data on coverage/consumption of iron‐containing supplements

Key informants indicated that national survey data are used for programme implementation and monitoring, managing the supply chain, and improving national policy (Supporting Information Table A6). Suggestions from key informants for additional information or indicators included asking about the dose of the supplement being consumed, the inclusion of other groups for whom iron‐containing supplementation programs exist (e.g., adolescent girls), and asking about reasons for poor adherence (e.g., side effects).

The key informant from Rwanda indicated that it would be helpful to have additional data to determine how many women suffer side effects from iron‐containing supplementation, while the informant from Myanmar suggested that coverage/consumption data should be used to identify geographical differences in iron‐containing supplement coverage and compliance to improve programme implementation and delivery.

### 3.2 Online survey

A total of 142 respondents from 52 countries completed the online questionnaire (Supporting Information Table A7). Fifty (35%) respondents from 21 countries worked for their government, while 40 (28%) worked for an NGO (Supporting Information Table A8). Three respondents did not report their country and are not included in the analysis.

#### 3.2.1 National guideline for iron‐containing supplement provision to adolescent girls, pregnant and/or lactating women

Thirty‐four respondents from 24 countries answered the question about whether there is an existing national guideline, policy, or protocol for iron‐containing supplement provision to adolescent girls, pregnant and/or lactating women. Eighteen (75%) countries have currently available guidance for iron‐containing supplement provision to adolescent girls, pregnant and/or lactating women (Supporting Information Table A9).

#### 3.2.2 Availability of iron‐containing supplements at health facilities

Thirty‐one respondents from 21 countries provided information on the availability of iron‐containing supplements at health facilities through ANC. Twenty (95%) reported that iron‐containing supplements are available; in 17 (81%) countries they are provided free of charge (Supporting Information Table A10).

#### 3.2.3 Coverage/consumption of iron‐containing supplements

An error in the online survey skipped the question of whether coverage/consumption of iron‐containing supplements was assessed in a national survey. However, in the follow‐up question on the timing of the most recent survey, respondents from 25 countries identified a nationally representative survey as the most recent survey assessing iron‐containing supplement coverage/consumption. In 24 of these countries, a national survey had been carried out between 2014 and 2018. For the 27 countries where a survey was not identified by respondents, Google Scholar, PubMed, and the WHO Micronutrients Database were searched to identify the most recent survey (Supporting Information Table A11).

#### 3.2.4 Assessment of iron‐containing supplement coverage/consumption in surveys

Less than half of the respondents (26 of 58, from 31 countries) were satisfied with the way in which iron‐containing supplement coverage/consumption is currently assessed in national surveys (Supporting Information Table A12). Of the 55% (*n* = 32) who responded “No” or “Other,” 24 (75%) provided open response suggestions on how questions assessing iron‐containing supplement coverage/consumption should be revised. These included shortening the recall period, including other beneficiary groups and inserting questions to assess if, and how, counselling for iron‐containing supplement consumption is provided (Supporting Information Table A13).

#### 3.2.5 Use of data on coverage/consumption of iron‐containing supplements

Thirty‐five respondents from 20 countries described data use (Supporting Information Table A14). The most common use of data collected on iron‐containing supplement coverage/consumption was in monitoring and evaluating programs (*n* = 19; 53%) and research (*n* = 7; 20%), followed by implementation (*n* = 5; 17%) and policy (*n* = 5; 17%).

### 3.3 Demographic and Health Surveys

Details of the number of days/months that iron‐containing supplements were consumed by pregnant women in their last pregnancy and the variables included in DHS data quality analysis are presented in Tables 1 and 2.

**Table 1:** Reported consumption of antenatal iron‐containing supplements from National Demographic and Health Surveys, by country

	Afghanistan		Colombia^a^		Myanmar		Tanzania	
	%	*n*	%	*n*	%	*n*	%	*n*
Coverage of any iron‐containing supplement consumption	89.3	8108^b^	95.2	8202^b^	98.0	3056^b^	98.0	5548^b^
Iron‐containing supplement consumption,^c^ mean days or months (±SE)	46.2 (±2.02)	18897	5.4 (±0.04)	9181	112.1 (±1.7)	3489	55.8 (±0.88)	6853
Iron‐containing supplement consumption by place ANC received, mean days or months (±SE)								
Public health facilities	46.2 (±2.53)	7354	5.2 (±0.05)	4652	117.1 (±1.85)	2168	55.7 (±0.96)	5762
Private health facilities	50.4 (±3.24)	2965	5.7 (±0.07)	4265	127.5 (±5.46)	262	58.4 (±6.14)	194

Abbreviations: ANC, antenatal care; SE, standard error of the mean.

^a^ Results for Colombia are in months.

^b^ Number of women who received or purchased any iron tablets/syrup.

^c^ Among those who reported receiving/purchasing iron supplements.

*Data Source*: Demographic and Health Surveys.

**Table 2:** Descriptive analysis of variables included in DHS data quality analysis, by country

	Afghanistan		Colombia		Myanmar		Tanzania	
Recall period, y	%	*n*	%	*n*	%	*n*	%	*n*
<1	30.5	5770	23.1	2143	22.9	798	29.0	1988
1 to <2	28.2	5324	22.8	2112	23.6	823	29.7	2038
2 to <3	21.9	4137	19.6	1819	19.5	680	18.9	1293
3 to <4	12.1	2287	18.3	1701	19.1	665	13.1	897
4 to <5	7.3	1379	16.2	1507	15.0	524	9.3	637
Number of ANC visits, mean (±SE)	1.9 (±0.07)	18897	7.2 (±0.07)	9281	4.8 (±0.14)	3489	3.7 (±0.04)	6853
No. ANC visits	%	*n*	%	*n*	%	*n*	%	*n*
<4	81.5	15078	8.7	793	40.7	1409	49.2	3353
≥4	18.5	3432	91.3	8337	59.4	2058	50.8	3467
No. ANC visits	%	*n*	%	*n*	%	*n*	%	*n*
<8	97.6	18069	56.9	5196	82.4	2857	98.8	6740
≥8	2.4	441	43.1	3934	17.6	610	1.2	80
Place ANC received	%	*n*	%	*n*	%	*n*	%	*n*
Public health facilities	71.2	7767	52.1	4699	89.1	2204	96.7	5857
Private health facilities	28.8	3140	47.9	4318	10.9	269	3.3	199
Maternal educational attainment	%	*n*	%	*n*	%	*n*	%	*n*
No education	82.7	15622	1.8	169	16.0	559	19.3	1324
Incomplete primary	6.1	1146	7.2	664	26.7	930	12.2	839
Complete primary	2.2	413	8.2	761	18.9	659	52.2	3576
Incomplete secondary	4.4	839	21.7	2015	27.5	959	5.3	362
Complete secondary	2.9	556	28.3	2624	2.5	88	10.1	689
Higher	1.7	321	32.8	3048	8.4	294	0.9	62

Abbreviations: ANC, antenatal care; SE, standard error of the mean.

*Data Source*: Demographic and Health Surveys.

#### 3.3.1 Descriptive analyses

Among the four countries, the highest reported consumption of antenatal iron among women who received or purchased any iron‐containing supplements was in Colombia (5.4 months) and Myanmar (112 days). Women in Afghanistan reported the lowest consumption of iron‐containing supplements during their last pregnancy (46 days). Similarly, the proportion of women who received at least four ANC visits during their last pregnancy was highest in Colombia (91%) and lowest in Afghanistan (18%). Maternal literacy was also lowest in Afghanistan, with 83% of women reporting no formal education. More than 60% of women in Colombia reported completing secondary school or higher.

#### 3.3.2 Data heaping

Data heaping was assessed descriptively for Afghanistan, Myanmar, and Tanzania (Table 3). Approximately 75% of women reported consuming iron tablets/syrup in multiples of 30 days: 0, 30, 60, 90, 120, 150, and 180. According to the key informants, tablets are distributed in packets of 10 in Afghanistan, and 30 in Myanmar and Tanzania. Therefore, data heaping around multiples of 10 and/or 30 is expected.

**Table 3:** Descriptive assessment of data heaping in reported days of iron tablet/syrup consumption by postpartum women in three^a^ DHS datasets

	Afghanistan		Myanmar		Tanzania	
Reported number of days	*N*	%	*N*	%	*N*	%
Don't know	855	4.6	55	1.6	112	1.6
0^b^	10326	56.1	423	12.2	1325	19.3
Multiples of 30 (i.e., 30, 60, 90)	3219	17.5	2289	65.8	3886	56.7
Other	4020	21.9	708	20.4	1525	22.3
Total	18420	100.0	3474	100.0	6848	100.0

Abbreviation: DHS, Demographic and Health Surveys.

^a^ Colombia is not included since antenatal iron‐containing supplement consumption was captured in months and not days.

^b^ Includes those who did not buy/receive iron tablets/syrup.

We also assessed data heaping among women who reported consuming iron tablets/syrup for at least one day, stratified by categories of recall period (Supporting Information Table A15). The proportion of women reporting iron‐containing supplement consumption in multiples of 30 days was high in Myanmar (75%) and Tanzania (70%), but much lower in Afghanistan (40%). Within each country, the number of days and the proportions reporting multiples of 30 were consistent across all recall period subgroups. Therefore, there does not appear to be bias due to recall of consumption over time.

We also compared the SE for each recall period category with the overall sample SE. Our hypothesis was that if the accuracy of women's recall of iron tablets/syrup consumption did not decline over time, the SE days/months of iron‐containing supplement consumption stratified by recall period would be similar to the overall SE days/months of iron‐containing supplement consumption. Using this approach, we did not observe any consistent trends in SE either across countries or across recall periods, and, therefore, cannot conclude that longer recall periods lead to deterioration of recall (Supporting Information Table A16).

#### 3.3.3 Linear regression

Results of linear regression analyses are presented in Table 4. For Myanmar and Tanzania, there is a significant (*p* < 0.05) inverse linear association between the current age of the child in months (i.e., recall period) and the number of days during which iron tablets/syrup were consumed. This association does not hold for Afghanistan and Colombia.

**Table 4:** Results of linear regression analyses between consumption of any iron‐containing supplement and recall period, number of ANC visits, and maternal educational attainment, by country

	Afghanistan		Colombia		Myanmar		Tanzania	
	Slope	*p*	Slope	*p*	Slope	*p*	Slope	*p*
Recall period	−1.220	0.1341	0.0123	0.6589	−2.406	0.0199	−1.859	0.0010
Number of ANC visits	3.771	<0.0001	0.3129	<0.0001	7.4447	<0.0001	5.3659	<0.0001
Maternal educational attainment	4.230	0.0016	0.2804	<0.0001	9.2025	<0.0001	3.3857	<0.0001
Maternal educational attainment controlling for ANC visits	3.3886	0.0054	0.1387	<0.0001	3.7789	<0.001	2.2063	0.0036

Abbreviation: ANC, antenatal care.

*Data Source*: Demographic and Health Surveys.

A significant (*p* < 0.001) positive linear association is present between the number of days/months during which iron‐containing supplements were consumed during pregnancy and the number of ANC visits for all four countries. Furthermore, there is a significant (*p* < 0.01), positive linear association between maternal educational attainment and the number of days/months during which iron‐containing supplements were consumed during pregnancy for all four countries. This association is attenuated, but still significant when adjusted for the number of ANC visits.

#### 3.3.4 Quality of iron‐containing supplement consumption data

As presented in Table 5, CV values across all categories of recall period are within the acceptable threshold of <0.166 recommended by Statistics Canada (Canadian Travel Survey Microdata User's Guide, 1997). CV values across categories of ANC visits and maternal educational attainment were also acceptable (data not shown).

**Table 5:** Quality assessment of iron‐containing supplement consumption data collected in DHS by recall period and country

	Afghanistan				Colombia				Myanmar				Tanzania			
	Mean days (95% CI)	SE	CV	*N*	Mean months (95% CI)	SE	CV	*N*	Mean days (95% CI)	SE	CV	*N*	Mean days (95% CI)	SE	CV	*N*
Recall period, y																
<1	46.4 (42.9–50.0)	2.15	0.046	5479	5.5 (5.3–5.6)	0.10	0.018	2136	114.6 (109.5–119.8)	3.12	0.027	790	59.3 (57.0–61.6)	1.39	0.024	1966
1 to <2	50.7 (43.8–57.7)	4.23	0.083	5100	5.4 (5.2–5.5)	0.09	0.017	2095	117.2 (112.8–121.6)	2.68	0.023	811	55.4 (52.9–58.0)	1.54	0.028	2018
2 to <3	40.5 (37.5–43.5)	1.80	0.083	3982	5.4 (5.2–5.5)	0.09	0.018	1801	111.3 (106.3–116.3)	3.03	0.023	670	53.8 (50.8–56.9)	1.84	0.028	1263
3 to <4	46.1 (38.4–53.8)	4.68	0.101	2152	5.3 (5.1–5.5)	0.12	0.022	1682	105.2 (99.9–110.6)	3.24	0.031	653	52.8 (49.9–55.6)	1.73	0.033	874
4 to <5	43.2 (38–48.5)	3.19	0.074	1328	5.7 (5.5–5.8)	0.10	0.017	1466	109.3 (103.1–115.6)	3.79	0.035	511	53.1 (48.8–57.5)	2.62	0.049	620

Abbreviations: CI, confidence interval; CV, coefficient of variation; SE, standard error of the mean.

*Data Source*: Demographic and Health Surveys (DHS).

## 4 DISCUSSION

Our study aimed to determine whether countries are collecting data on antenatal iron‐containing supplement consumption, and if so, how these data are being collected and used. We also assessed whether we could identify any differences in the quality of data derived from current DHS questions on antenatal iron‐containing supplementation across the five‐year recall period. Although we found no conclusive evidence of deteriorating data quality by period of recall, feedback gathered from key informants and online survey participants highlight the need identified by the WHO–UNICEF TEAM group, that there is demand for a revised indicator and measurement guidance for coverage of iron‐containing supplementation during pregnancy.

Both KII and online survey participants, who came from a range of affiliations across government, nongovernmental and United Nations organisations, reported similar challenges with antenatal iron‐containing supplement data collection in large‐scale surveys; less than half the respondents were satisfied with the current methodology. These concerns included: (i) long recall period; (ii) lack of data on counselling, including for possible side‐effects of iron‐containing supplement consumption for pregnant women; (iii) need for data on other beneficiary groups; and (iv) need for data on supply‐side issues, such as stock‐outs, which affect the access of pregnant women to iron‐containing supplements.

Evidence of number heaping in the reported number of days of iron‐containing supplement consumption was not entirely unexpected given that tablets are often distributed in packs of 10 or 30 and the prompts included in the DHS questions require the enumerator to show the iron‐containing tablets/syrup in packs or bottles (DHS, 2018). In attempting to recall supplements consumed several months to several years ago, women are also likely to round up their estimates in multiples of tens. Furthermore, the lack of discernible differences in data heaping or in quality of data (as measured by CV) across categories of recall period indicates that time since delivery was not likely a driver in this data heaping or quality. However, since there is no way to verify the veracity of women's recall of iron‐containing supplement consumption in DHS data, this finding should be interpreted with caution.

The low CV values we observed in the DHS analyses indicate acceptable dispersion of data; however, CV does not measure the presence of any systematic biases in survey data which might affect the estimate (*Canadian Travel Survey Microdata User's Guide*, 1997). Although Kanyangarara et al. (2019) reported on actual iron‐containing supplement receipt at exit interviews in comparison to reported consumption, they did not report CV as a measure of dispersion across data for comparison. Therefore, we cannot confirm whether CV was an appropriate choice to assess data quality across the recall period in the absence of validation data.

There is a need for indicator and question validation research that compares a gold standard measure of actual consumption to recall across time. Many of our key informants and online survey respondents expressed concern about the accuracy of the 5‐year recall period for ANC in the DHS. The recommended GNMF indicators reduced the recommended recall to a maximum of 2 years (WHO, 2017; WHO, 2018a). In a nine‐country analysis using data from service provision assessments, Kanyangarara et al. (2019) found that clients participating in an exit interview immediately following their ANC visit were more likely to report receipt when no supplement was given (false positive) than to report no receipt when supplements were actually given (false negative). Kanyangarara et al. (2019) also assessed the validity of self‐reported receipt of iron‐containing supplements among a smaller sample of women 1–2 years postpartum in Nepal and found that sensitivity was slightly higher while specificity was much lower in client exit interviews compared to observed service. This could mean there is a tendency, at least in this population, to overestimate receipt as time since the event increases.

Across all four countries with DHS analyses, reported consumption of iron‐containing supplements was associated with maternal educational attainment independent of the number of ANC visits. Education level may affect the consistency, quality, and validity of antenatal iron‐containing supplement consumption data. This finding is similar to that of Kanyangarara et al. (2019), who reported that higher maternal education was associated with poorer agreement between observed receipt of iron‐containing supplementation and recall of consumption. In our analyses, we could not verify recall with direct observation, so we cannot confirm whether there are or are not agreements between educational attainment and data quality or which direction they might take if present.

Because there are few studies addressing quality of recall across time for iron‐containing supplementation, we looked for studies that evaluated the quality of recall for other interventions during pregnancy, birth, and early postpartum. Sundermann et al. (2017) found that overall agreement was >70% for preconception nonsteroidal anti‐inflammatory drugs exposure when comparing prepregnancy direct diary records with recall during the first trimester of pregnancy. Amissah et al. (2017) found that overall, maternal recall of breastfeeding duration was valid 6 years after childbirth, while Li et al. (2005) found that validity and reliability of breastfeeding initiation and duration were better when the recall period was less than 3 years compared to 3–5 years. Agreement between the observed prevalence of exclusive breastfeeding at 3 months postpartum, and a 12‐month postpartum survey that asked about the prevalence of breastfeeding practices at 3 months postpartum was also found to be high (Schneider et al., 2020). Finally, Hu et al. (2019) found >90% agreement between clinical records of vaccinations given to children in the previous year and maternal recall. These studies comparing direct observation with recall across several different timeframes report variable validity depending on the intervention. The generally better recall was associated with shorter recall periods, which is consistent with the concerns expressed by our study respondents about the long recall period in survey questions.

The positive correlations observed between the number of ANC visits and the number of iron‐containing supplements consumed align with findings from previous studies (Gopalakrishnan et al., 2019; Nguyen et al., 2017; Nisar et al., 2014; Wendt et al., 2015). Referring to ANC visits as a method of refining probes may be useful in improving recall and reporting accuracy but more research is needed about how this might affect the accuracy of recall.

Informants suggested that surveys should include questions about counselling on proper supplement use and adherence. The effectiveness of antenatal counselling on changing maternal behaviour is well‐established (Nguyen et al., 2019, 2017; Nisar et al., 2014). However, DHS does not capture data on antenatal nutrition counselling and so we were not able to assess whether iron‐containing supplement consumption is associated with nutrition counselling during pregnancy.

Considering the limitations of current antenatal iron‐containing supplement coverage questionnaires and desire for improved questions and indicators, future research should more precisely quantify the quality of antenatal iron‐containing supplement consumption recall data by: (i) assessing the potential impact on sample size and results with the shorter recall period of the DHS‐8 antenatal iron‐containing supplement consumption indicator (DataDENT) (ii) identifying studies in which observed receipt or consumption could be compared to recall across 0–2 years; (iii) exploring possibilities for integrating nutrition counselling indicators into large‐scale surveys and determining how counselling might affect postnatal iron‐containing supplement consumption recall; and (iv) evaluating data collection and questions from the mothers’ perspective to optimise recall and the use of probes.

## 5 CONCLUSION

Although our findings were inconclusive in terms of whether antenatal iron‐containing supplement consumption data should be collected over a 5‐year or shorter recall period, the concerns consistently expressed by respondents and the findings of a study on reduced quality of recall when assessed 1–2 years after giving birth (Kanyangarara et al., 2019) support the GNMF‐proposed indicator, which has a recall period of not more than 2 years. However, this will have implications for planned sample sizes of large‐scale, population‐based surveys, such as the DHS. Further research is needed to enhance survey methodology and determine how the quality of antenatal iron‐containing supplement consumption recall data can be improved to more accurately quantify supplement distribution and consumption. The WHO–UNICEF TEAM group will continue to seek evidence comparing actual to recall data to inform the finalisation of a global indicator and operational guidance to help countries collect and report on this indicator as part of the GNMF.

## CONFLICT OF INTERESTS

The authors declare that there are no conflict of interests.

## AUTHOR CONTRIBUTIONS

*Conceptualisation*: Sara Wuehler, Kuntal K. Saha, and Rebecca Heidkamp. *Methodology*: Aatekah Owais, Sara Wuehler, Kuntal K. Saha, and Rebecca Heidkamp. *Formal analysis*: Aatekah Owais. *Investigation*: Aatekah Owais. *Data curation*: Aatekah Owais. *Writing—original draft preparation*: Aatekah Owais. *Writing—review and editing*: Sara Wuehler, Kuntal K. Saha, Rebecca Heidkamp, Vrinda Mehra, Lisa M. Rogers, and Lynnette M. Neufeld. All authors made critical contributions to the concept, revisions to the manuscript, and approved the final version.

## Supporting information

Supporting information.

## Data Availability

Data available upon request from corresponding author.
