# Supplementary material for: Critical assessment of the current indicator for antenatal iron‐containing supplementation coverage: Insights from a mixed‐methods study
Source: Matern Child Nutr. 2022 Jan 28;18(2):e13314. doi: 10.1111/mcn.13314 (PMC8932708; doi:10.1111/mcn.13314)
Supplement: Supplementary file 1 — Supporting information. [file MCN-18-e13314-s001.docx]

# Web Appendix

## Table A 1: Key informant interview guide for WHO-UNICEF TEAM exploratory analysis of iron supplementation data collection and usage

| 1. Please indicate the nature of your work in relation to nutrition and supplement for pregnant women: 2. Policy development 3. Program development 4. Program implementation 5. Program surveillance 6. Procurement/Distribution 7. Other (please specify) 8. Please indicate the type of organization at which you work: 9. Government/ministry 10. Private agency 11. NGO 12. UN 13. Other (please specify) 14. Which country are you based in? 15. Is coverage/consumption of iron supplements assessed in your country’s national nutrition or other large-scale survey? Please consider this for supplements with just iron, iron plus folic acid OR with iron and multiple other micronutrients. 16. When was the most recent survey which assessed iron supplements coverage/consumption in your country?     1. Could you share the questions that were used/asked in that survey?     2. If more than one large-scale survey in your country collects data on iron supplements coverage/consumption, could you also share those questions? 17. Are you satisfied with the way iron supplement coverage/consumption is assessed in these surveys?     1. If no, how would you revise these questions?     2. What else about iron supplements would be useful to know? 18. How do you use the iron supplement coverage/consumption data collected in the above surveys?     1. If data are not used currently, how would you like to use them, or how would you recommend that they be used?     2. If 6b offers suggestions: How would you use additional information that you mentioned above? 19. Is there a national guideline or policy or protocol available for iron supplement provision to adolescent girls, pregnant and/or lactating women or does any other political administrative level have such a guideline?     1. If yes, what is its source (e.g. federal health agency, WHO)?     2. When was the guideline established or validated in your country?     3. Has it been updated since then?     4. If yes, what year was it updated? 20. Are iron supplements available at health facilities where pregnant women receive antenatal care (i.e. first point of contact between a pregnant woman and a health care provider)?     1. Is it provided free or at charge or both?     2. If at charge, what is the cost per strip/packet (in local currency)?     3. How many tablets are included in one strip/packet?     4. If not available at health facilities, where do women obtain iron supplements (e.g. prescription at a pharmacy, pharmacy without a prescription, NGOs, women’s group, school, workplace)? 21. Are iron supplements provided for another subset of the population (e.g. adolescent girls)?     1. If yes, whom are they provided for?     2. Is it provided free or at charge?     3. If at charge, what is the cost per strip/packet (in local currency)?     4. How many tablets are included in one strip/packet? 22. How is the supply chain for iron supplements managed by:     1. Government?     2. NGOs?     3. Private sector? 23. Is the budget for iron supplement procurement and disbursement allocated in the federal budget?     1. If no, what is the source (or what are the sources) of funding for iron supplement procurement and disbursement? |
| --- |

## Table A 2: Data quality guidelines used to assess Demographic and Health Survey data

| **Quality level of estimate** | **Guidelines** |
| --- | --- |
| 1) Acceptable | Low CV in the range of **0.0% to 16.5%** |
| 2) Marginal | High CV in the range of **16.6% to 33.3%** |
| 3) Unacceptable | Very high CV in excess of **33.3%** |
| Source: [Statistics Canada](http://www23.statcan.gc.ca/imdb-bmdi/document/3810_DLI_D1_T22_V8-eng.pdf#page=96); CV: Coefficient of variation | |

## Table A 3: Summary of findings from key informant interviews

| **Country** | **Most recent survey** | **Iron supplementation questions asked** | **Satisfied with iron supplementation questions** | **How are survey data used** | **National iron supplementation guideline** | **Iron supplements available at health centres** | **Supplements provided to group other than pregnant women** |
| --- | --- | --- | --- | --- | --- | --- | --- |
| Afghanistan | DHS 2015 | Those used in DHS | Yes | Program implementation, national policy and proposals/reporting | Yes | Yes, free of charge | Iron supplements in school for adolescent girls 10–19 years |
| Colombia | 1. DHS 2015  2. HMIS for pregnant women using ANC service  3. Quality of Life (QoL) Survey 2017 | DHS: Those used in DHS HMIS: Indicates if iron supplements were prescribed during ANC visit QoL: 1. Are you pregnant?  2. If yes, did you receive a prescription for iron, FA or calcium supplements? | No, as no information captured on compliance, or dose received and consumed by pregnant women | Monitor coverage of iron supplementation | Yes | Yes, free of charge | - Iron supplements for < 6 months if mother had low gestational weight - MNP for 6–24 months - Iron and vitamin A supplements for children aged 24–59 months  - Iron supplements for adolescents if diagnosed with anaemia |
| Guatemala | National Maternal and Child Health Survey 2015 | Similar to those used in DHS | No, as only receipt of iron supplements is captured, not consumption | Forecast procurement and identify areas of concern | Yes | Yes, free of charge | - Sprinkles for children 0-5 years - MNP tablets for children aged 6–10 years and lactating women |
| Japan | No national survey for iron supplements, but National Health and Nutrition Survey is conducted annually | No, but questions asked about current drug consumption, including for anaemia treatment (regardless of whether prescribed or purchased over-the-counter): Do you consume/take a drug for treatment of anaemia (iron)? Yes or No. | Not applicable | Not applicable | No national policy, but Japan’s obstetric/ gynaecological medical association has a guideline for iron/folic acid supplementation for pregnant women | Most of the cost is covered by Japan’s universal national health insurance, but pregnant women have to pay 20–30% | No universal supplementation for any subset of population |
| Myanmar | Myanmar Micronutrient and Food Consumption Survey 2017–2018 | Have you ever received iron supplements from the health care provider? Do/Did you take the iron supplements provided by the health provider? | Not sure, as analysis in not yet complete | Not used currently | Yes | Yes, free of charge | Iron supplements in school for adolescent girls aged 10–19 years |
| Niger | DHS 2012 | Those used in DHS | Not answered | Not answered | Yes | Yes, both free and at charge | Adolescent girls aged 10–19 years |
| Rwanda | DHS 2014–2015 | Those used in DHS | No, because women are not told how to take iron supplements or told of iron-rich foods available in the community | Not used currently | No, but it is under development | Yes, free of charge | MNP to children aged 6–24 months |
| Tanzania | National Nutrition Survey, 2018 | Not shared | No, as questions are retrospective, and it is unclear whether women are remembering correctly or just guessing | Improve national policy and programme implementation | Yes | Yes, free of charge | Yes, adolescent girls via pilot program in Simiyu Region |
| ANC: Antenatal care; DHS: Demographic and Health Survey; HMIS: Health Management Information System; MNP: Micronutrient Powders | | | | | | | |

## Table A 4: Beneficiaries included in national guidelines for iron and/or micronutrient supplement provision in countries in which key informant interviews were completed

| **Country** | **National guideline beneficiaries** | **Iron supplement formulation** |
| --- | --- | --- |
| *Anaemia prevalence > 40%* | | |
| Tanzania | PW | Not mentioned |
| Myanmar | PW and adolescent girls 10–19 years | Not mentioned |
| Niger | PW and adolescent girls 10–19 years | Not mentioned |
| *Anaemia prevalence 20–39.9%* | | |
| Colombia | PW, infants < 24 months^*^ and adolescents | 60 mg |
| Japan | No national guideline | Not applicable |
| *Anaemia prevalence < 20%* | | |
| Rwanda | Children 6–24 months | Not applicable |
| Guatemala | PLW and children 0–10 years | Not mentioned |
| Afghanistan | PW and adolescent girls 10–19 years | Not mentioned |
| ^*^Iron supplements for < 6 months if low gestational weight at birth; MNP for 6–24 months; iron and vitamin A supplements for children 24–59 months.  MNP: micronutrient powder; PW: pregnant women; PLW: pregnant and lactating women. | | |

## Table A 5: Antenatal iron supplementation indicator used in countries in which key informant interviews were completed

| **Country** | **Indicator definition** | **Denominator** | **Data source** |
| --- | --- | --- | --- |
| *Anaemia prevalence > 40%* | | | |
| Tanzania | Took none/any/<60/  60–89/90+ iron tablets/syrups | Women aged 15–49 with a live birth in the five years preceding the survey | National Nutrition Survey 2018 |
| Myanmar | Data not analysed | NA | Myanmar Micronutrient and Food Consumption Survey 2017–2018 |
| Niger | Took none/any/<60/  60–89/90+ iron tablets/syrups | Women aged 15–49 with a live birth in the five years preceding the survey | DHS 2012 |
| *Anaemia prevalence 20–39.9%* | | | |
| Colombia | Number of **months** iron consumed | Women aged 13–49 with a live birth in the five years preceding the survey | DHS 2015 |
| Japan | Distribution and mean (± SD) of haemoglobin | Women aged 20 or older, irrespective of birth history | National Health and Nutrition Survey 2015 |
| *Anaemia prevalence < 20%* | | | |
| Rwanda | Took none/any/<60/  60–89/90+ iron tablets/syrups | Women aged 15–49 with a live birth in the five years preceding the survey | DHS 2014–2015 |
| Guatemala | Took none/any/<60/  60–89/90+ iron tablets/syrups | Women aged 15–49 with a live birth in the five years preceding the survey | National Maternal and Child Health Survey 2015 |
| Afghanistan | Took none/any/<60/  60–89/90+ iron tablets/syrups | Women aged 15–49 with a live birth in the five years preceding the survey | DHS 2015 |

## Table A 6: Key results on collection and use of data on coverage/consumption of iron supplements

| **Country** | **Nutrition survey conducted in past 5 years** | **Iron supplement questions used** | **Satisfied with iron supplement questions** | **How are survey data used** |
| --- | --- | --- | --- | --- |
| *Anaemia prevalence > 40%* | | | | |
| Tanzania | 1. NNS 2018  2. DHS 2015–2016 | Not shared, but those used in NNS 2014 are similar to those used in DHS | No | Improve national policy and program implementation |
| Myanmar | 1. Myanmar Micronutrient and Food Consumption Survey (MMFCS) 2017–2018  2. DHS 2015–2016 | DHS: those used in DHS  MMFCS:  1. Have you ever received iron supplements from the health care provider?  2. Do/Did you take the iron supplements provided by the health provider? | Not sure, as data analysis is pending | Not used currently |
| Niger | DHS 2012 | Those used in DHS | Not answered | Not answered |
| *Anaemia prevalence 20–39.9%* | | | | |
| Colombia | 1. DHS 2015  2. HMIS  3. Quality of Life (QoL) Survey 2017 | DHS: those used in DHS HMIS: indicates if iron supplements were prescribed during ANC visit QoL:  1. Are you pregnant?  2. If yes, did you receive a prescription for iron, folic acid or calcium supplements? | No | Monitor coverage of iron supplement |
| Japan | Annual National Health and Nutrition Survey | Not applicable | Not applicable | Not applicable |
| *Anaemia prevalence < 20%* | | | | |
| Rwanda | DHS 2014–2015 | Those used in DHS | No | Not used currently |
| Guatemala | 1. National Maternal and Child Health Survey 2015  2. DHS 2014–2015 | Those used in DHS | No | Forecast procurement and identify areas of concern |
| Afghanistan | 1. Afghanistan Health Survey 2018  2. DHS 2015  3. Afghanistan Health Survey 2015 | AfHS: similar to those used in DHS  DHS: those used in DHS | Yes | Program implementation, national policy and proposals/reporting |

## Table A 7: List of online survey respondents’ countries

| **Country** | **N** | **%** |
| --- | --- | --- |
| Afghanistan* | 2 | 1.4 |
| Albania | 1 | 0.7 |
| Australia | 1 | 0.7 |
| Bangladesh | 1 | 0.7 |
| Belgium | 2 | 1.4 |
| Canada | 3 | 2.1 |
| Chile | 1 | 0.7 |
| China | 2 | 1.4 |
| Colombia* | 1 | 0.7 |
| Cote d’Ivoire | 1 | 0.7 |
| Ethiopia | 5 | 3.5 |
| Fiji | 1 | 0.7 |
| Germany | 1 | 0.7 |
| Ghana | 6 | 4.2 |
| Greece | 1 | 0.7 |
| India | 23 | 16.2 |
| Indonesia | 2 | 1.4 |
| Iran | 3 | 2.1 |
| Iraq | 1 | 0.7 |
| Japan* | 3 | 2.1 |
| Jordan | 1 | 0.7 |
| Kenya | 10 | 7.0 |
| Lebanon | 2 | 1.4 |
| Lesotho | 1 | 0.7 |
| Malawi | 1 | 0.7 |
| Malaysia | 1 | 0.7 |
| Mexico | 2 | 1.4 |
| Moldova | 1 | 0.7 |
| Mozambique | 1 | 0.7 |
| Nepal | 1 | 0.7 |
| Nigeria | 5 | 3.5 |
| Oman | 1 | 0.7 |
| Pakistan | 8 | 5.6 |
| Peru | 4 | 2.8 |
| Philippines | 5 | 3.5 |
| Portugal | 1 | 0.7 |
| Serbia | 1 | 0.7 |
| South Africa | 1 | 0.7 |
| South Sudan | 1 | 0.7 |
| Sri Lanka | 2 | 1.4 |
| Sudan | 1 | 0.7 |
| Syria | 1 | 0.7 |
| Thailand | 1 | 0.7 |
| UAE | 1 | 0.7 |
| Uganda | 3 | 2.1 |
| United Kingdom | 1 | 0.7 |
| Uruguay | 2 | 1.4 |
| USA | 14 | 9.9 |
| Venezuela | 1 | 0.7 |
| Vietnam | 1 | 0.7 |
| Zambia | 1 | 0.7 |
| Zimbabwe | 2 | 1.4 |
| Other | 2 | 1.4 |
| **Total** | **142** | **100.0** |
| Sample sizes differ according to the total number who responded to each question  *KII also completed | | |

## Table A 8: Demographic characteristics of online survey respondents

| **Fields of work occupied by respondents** | **N** | **%** |
| --- | --- | --- |
| Academic/research | 27 | 19.1 |
| Dietician/nutritionist | 5 | 3.5 |
| Policy development | 18 | 12.8 |
| Procurement/distribution | 4 | 2.8 |
| Program development | 29 | 20.6 |
| Program implementation | 32 | 22.7 |
| Program surveillance | 18 | 12.8 |
| Other | 8 | 5.7 |
| **Total** | **141** | **100.0** |
| **Type of organization employing respondents** | **N** | **%** |
| Academia | 17 | 11.9 |
| Government/ministry | 50 | 35.0 |
| NGO | 40 | 28.0 |
| Private agency | 13 | 9.1 |
| UN | 17 | 11.9 |
| Other | 6 | 4.2 |
| **Total** | **143** | **100.0** |
| Sample sizes differ according to the total number who responded to each question | | |

## Table A 9: Status of the national guideline for iron supplement provision in countries mentioned in the online survey

| **Country** | **Anaemia prevalence among women of reproductive age** | **Guideline status** |
| --- | --- | --- |
| *Anaemia prevalence > 40%* | | |
| India | 51* | Available |
| Nigeria | 50* | Available |
| Ghana | 47.7** | Available |
| Pakistan | 43.5** | Available |
| *Anaemia prevalence 20 – 39.9%* | | |
| Malawi | 35.3** | Available |
| Nepal | 35** | Available |
| Syria | 34* | Not available |
| Iran | 33.4** | Available |
| Sri Lanka | 33* | Available |
| Thailand | 32* | Available |
| Fiji | 31* | Available |
| Moldova | 27.9** | Available |
| Kenya | 27* | Available |
| Indonesia | 26* | Not available |
| Venezuela | 24* | Not available |
| Peru | 21** | Available |
| Uruguay | 21* | Available |
| Japan | 20.8** | Not available |
| *Anaemia prevalence < 20%* | | |
| China | 19.9** | Not available |
| Portugal | 18* | Available |
| Philippines | 16* | Under development |
| USA | 13* | Available |
| Afghanistan | 12.9** | Available |
| Mexico | 11.8** | Available |
| Sources: **^*^**[The World Bank](https://data.worldbank.org/indicator/SH.ANM.ALLW.ZS), accessed 30 August 2019; **^*^**^*^[WHO Micronutrients database](https://www.who.int/teams/nutrition-and-food-safety/databases/vitamin-and-mineral-nutrition-information-system), accessed 4 December 2018. | | |

## Table A 10: Availability of iron supplements at health facilities in countries mentioned in the online survey

| **Iron supplements available at health facility** | **% (n)** |
| --- | --- |
| Yes, free of charge | 81.0 (17) |
| Yes, at a charge | 0.0 (0) |
| Yes, both | 14.3 (3) |
| No | 4.8 (1) |
| Total | 100.0 (21) |

## Table A 11: List of countries, year and name of most recent survey assessing iron supplement coverage/consumption

| **Country** | **Year of survey** | **Name of survey** | **Reported by online survey respondent** |
| --- | --- | --- | --- |
| *Anaemia prevalence > 40%* | | | |
| Mozambique^*€^ | 2018 | Mozambique Demographic and Health Survey (DHS) |  |
| Cote d’Ivoire^*€^ | 2016 | Multiple Indicator Cluster Survey (MICS) |  |
| India^¥^ | 2015–2016 | National Family Health Survey (NFHS-4) | Yes |
| Nigeria^¥^ | 2013 | Nigeria Demographic and Health Survey | Yes |
| Bangladesh^€^ | 2014 | Bangladesh Demographic and Health Survey | Yes |
| Ghana^€^ | 2017 | Ghana Micronutrient Survey 2017 (GMS 2017) | Yes |
| Pakistan^€^ | 2017 | Pakistan Demographic and Health Survey | Yes |
| *Anaemia prevalence 20–39.9%* | | | |
| Jordan^*€^ | 2019 (ongoing) | Jordan National Micronutrient & Nutrition Survey |  |
| Malawi^€^ | 2015–2016 | Malawi Micronutrient Survey | Yes |
| Nepal^€^ | 2016 | National Micronutrient Survey | Yes |
| South Sudan^*¥^ | 2018 | Standardized Expanded Nutrition Survey (SENS) |  |
| Syria^¥^ | 2017 (ongoing) | SMART Survey | Yes |
| Zambia^*¥^ | 2014 | Zambia Food Consumption and Micronutrient Status Survey |  |
| Iran^€^ | 2011–2015 | National Integrated Micronutrient Survey (NIMS-II) | Yes |
| Oman^€^ | 2017 | National Nutrition Survey | Yes |
| Sri Lanka^¥^ | 2016 | Sri Lanka Demographic and Health Survey | Yes |
| Uganda^*€^ | 2016 | Uganda Demographic and Health Survey (DHS) |  |
| Colombia^*€^ | 2015 | National Survey of the Nutritional Situation of Colombia |  |
| Thailand^¥^ | 2014 | Thai National Health Examination Survey | Yes |
| Fiji^¥^ | 2014–2015 | National Nutrition Survey | Yes |
| Sudan^*¥^ | 2010 | Multiple Indicator Cluster Survey (MICS) |  |
| Iraq^*¥^ | 2018 | Multiple Indicator Cluster Survey (MICS) |  |
| UAE^*¥^ | 2009–2010 | Research study^**^ |  |
| Moldova^€^ | 2005 | Moldova Demographic and Health Survey | Yes |
| Lesotho^*€^ | 2014 | Lesotho Demographic and Health Survey (DHS) |  |
| Kenya^¥^ | 2014 | Kenya Demographic and Health Survey | Yes |
| Serbia^*¥^ | 2013 | Serbian National Health Survey |  |
| Zimbabwe^*€^ | 2018 | National Nutrition Survey |  |
| Indonesia^€^ | 2018 | MARS (Medication Adherence Rating Score) of Iron Supplementation | Yes |
| South Africa^*¥^ | 2012 | South African National Health and Nutrition Examination Survey |  |
| Malaysia^*¥^ | 2014 | Malaysian Adult Nutrition Survey (MANS) |  |
| Venezuela^¥^ | 2015 | Encuesta de Consumo Diario de alimentos y Frecuencia de Consumo de Alimentos | Yes |
| Vietnam^*¥^ | 2009–2010 | General Nutrition Survey |  |
| Lebanon^*€^ | 1996 | Lebanon Mother and Child Health Survey |  |
| Peru^€^ | 2018 | Encuesta Demográfica y de Salud Familiar | Yes |
| Uruguay^¥^ | 2018 | Not mentioned | Yes |
| Japan^€^ | 2011 (ongoing) | The Japan Environment and Children’s Study | Yes |
| *Anaemia prevalence < 20%* | | | |
| China^*€^ | 2015 | China Health and Nutrition Survey |  |
| Albania^*€^ | 2017 | Albania Demographic and Health Survey (DHS) |  |
| Portugal^*¥^ | 2015–2016 | National Food, Nutrition and Physical Activity Survey |  |
| Ethiopia^*€^ | 2016 | Ethiopia Demographic and Health Survey (DHS) |  |
| Belgium^*¥^ | 2014–2015 | Belgian National Food Consumption Survey |  |
| Germany^*¥^ | 2005–2007 | German National Nutrition Survey II |  |
| Greece^¥^ | 2013–2014 | Not mentioned | Yes |
| Philippines^¥^ | 2018 (report pending) | Maternal Health and Nutrition and Infant and Young Child Feeding Surveys | Yes |
| Chile^*¥^ | 2010–2011 | Chile National Food Consumption Survey |  |
| United Kingdom^*¥^ | 2008–2017 | National Diet and Nutrition Survey |  |
| USA^¥^ | 2015–2016 | National Health and Nutrition Examination Survey (NHANES) | Yes |
| Afghanistan^€^ | 2015 | Afghanistan Demographic and Health Survey | Yes |
| Mexico^€^ | 2016 | Not mentioned | Yes |
| Australia^*¥^ | 2011–2013 | Australian Health Survey |  |
| Canada^*€^ | 2015 | Canadian Community Health Survey, Nutrition |  |
| ^*^Survey identified via Google Scholar, PubMed and VMNIS database; Anaemia prevalence among women of reproductive age sources: ^€^[VMNIS database](https://www.who.int/vmnis/database/en/), accessed 4 December 2018; ^¥^[The World Bank](https://data.worldbank.org/indicator/SH.ANM.ALLW.ZS), accessed 30 August 2019.  ^**^Ng, S. W., Zaghloul, S., Ali, H., Harrison, G., Yeatts, K., El Sadig, M., & Popkin, B. M. (2011). Nutrition transition in the United Arab Emirates. Eur J Clin Nutr, 65(12), 1328-1337. doi:10.1038/ejcn.2011.135 | | | |

## Table A 12: Satisfaction with how iron supplement coverage/consumption is assessed in national surveys

| **Satisfied with iron supplement coverage/consumption assessment** | **% (n)** |
| --- | --- |
| Yes | 44.8 (26) |
| No | 37.9 (22) |
| Other: |  |
| - Not applicable/no data | 12.1 (7) |
| - Recall bias | 1.7 (1) |
| - Lack of antenatal nutrition counselling and information on stock-outs | 1.7 (1) |
| - Lack of qualitative data | 1.7 (1) |
| **Total** | **100.0 (58)** |

## Table A 13: Respondents’ suggestions on how questions assessing iron supplement coverage/consumption should be revised in national surveys

| **Country** | **Suggestions for revision** |
| --- | --- |
| Afghanistan | “It should be tracked through the health system surveillance on regular bases rather than waiting for a large-scale survey after 5–10 years.” |
| Canada | “Need better data around consumption of >90 supplements; >150 supplements; KAP.” |
| Germany | “A lot of times we do not know compliance and length of duration people take the supplements. This is important though. Equally important is to know whether people eat an iron-rich diet. Did you take the iron pills on a daily basis? Did you encounter side effects? For how long did you take the iron supplements? How many times are you eating meat per week? How many times are you eating legumes per week?” |
| Ghana | “DHS questions are based on recall because it selects mothers who have had a pregnancy/birth in the five years preceding the survey. How would mothers remember the number of IFA tablets consumed for the entire duration of pregnancy five years ago? I seriously think when it comes to IFA intake the question should be posed to currently pregnant mothers to minimize recall bias.” |
| India | “Only pregnant women are covered. Other beneficiary groups are not covered. There is no dedicated survey for anaemia in India.” |
| India | “The inclusion of all vulnerable groups. The following questions can be added. Total number of IFA distributed. Follow-up by ANM/ASHA.” |
| India | “Bio-markers for compliance should be used.” |
| Iran^*^ | “The safety of parenteral versus oral iron administration. For anaemia, systematic review and meta-analysis.” |
| Japan | “The questions ask: "Do you take any iron supplementation?" followed by a question: "If so, what?" As the answer is by text, the analysis is difficult and drug dose may not be available for some cases.”^1^ |
| Kenya | “Include questions specific to adolescent girls 10–19 years of age.” |
| Kenya | “Reduce the recall period. Add additional questions to better inform programming.” |
| Kenya | “To include adolescent coverage at the ANC.” |
| Kenya | “Include questions on: sources of the supplements; total amounts of IFA consumed (e.g. data on g/day and days consumed); time of start of IFA use; data on reasons for low compliance/late start to IFA use; content disseminated during ANC counselling sessions.” |
| Malawi | “There should be a validation also. Govt should also make a record on consumption data.” |
| Mexico | “No estuve satisfecha, por que desafortunadamente no logramos captar bien frecuencia de consumo, cantidad ni veces por día, quizás por mala capacitación. Pero yo incluiría las mismas preguntas, e incluiría meses (tiempo) totales de consumo.” |
| Nigeria | “I would like for a survey to be carried out and used as a baseline for future surveys.” |
| Pakistan | “Measurement and dietary habit calculations.” |
| Peru | “Pregunta para complementar: ‘En la última semana, ¿cuántos días consumió los suplementos?’” |
| Peru | “Se les realiza el dosaje de hemoglobina.” |
| Thailand^*^ | “I am doing my PhD (I am 70 years old) on weekly iron supplementation + health literacy approach model among women of reproductive age in factories.” |
| Uruguay | “Madres de niños de 12 a 59 meses, los últimos 6 meses ¿han recibido chispitas nutricionales?” |
| USA | “I would add regarding what is counselled on for anaemia (if counselling takes place), and have responses to find out if the providers actually counsel on benefits, on side effects, etc.” |
| USA^**^ | “In my experience working in antenatal clinics in Malawi 2013–2016, the public clinics were frequently out of stock for IFA. Would prefer a system of monitoring supplies provided to clinics and proportion of women actually receiving it monthly, and tracking how many days of the pregnancy that supplements were provided for. Women attend ~ 4 visits, and if the clinic had supplies, they provided 28 days worth.” |
| *Not mentioned* | “I am not telling that the questions are not good enough to capture the information. I am just opined that the coverage for IFA is still low in spite of best education and BCC efforts. However, the coverage (just receipt of tablets) is somewhat satisfactory but mandatory consumption of 90 or 100 tablets during pregnancy was low and during lactation was very low. Very difficult to assess compliance of consumption of the IFA tablets. However, we are also asking some additional questions like, if consumed IFA tablets, how many? and please show the remaining tablets with you (pregnant/lactating women). If the balance tablets matching with the consumed tablets, then we are taking the compliance of consumption is good.” |
| ^*^Comment excluded from analysis since it does not seem to relate to the question asked.  ^**^Appears to be referencing work in Malawi, although self-reported as being in the USA.  ANC: Antenatal care; ANM/ASHA: Auxiliary Nurse Midwife/[Accredited Social Health Activist](https://en.wikipedia.org/wiki/Accredited_Social_Health_Activist); BCC: Behaviour Change Communication; DHS: Demographic and Health Survey; IFA: Iron and Folic Acid; KAP: Knowledge, Attitude, and Practices | |

Note: verbatim responses edited slightly for clarity.

## Table A 14: Details of how data collected on iron supplement coverage/consumption is used

| **Country** | **Use of iron supplementation/coverage data** | **Type of Usage** |
| --- | --- | --- |
| Afghanistan | “For programme planning.” | Monitoring |
| Canada | “To monitor progress in iron supplementation coverage at national level.” | Monitoring |
| Canada | “Monitoring and evaluation of programmes.” | Monitoring |
| Fiji | “Assess coverage and informing policy decisions.” | Monitoring |
| Germany | “To inform project/programme design, meaning to see if we need to include iron supplementation into our projects.” | Research |
| Ghana | “I use it for advocacy and lobbying tool for anaemia control programs to worse hit areas.” | Other |
| Ghana | “To estimate the coverage of iron supplement/coverage in my region.” | Monitoring |
| Ghana | “To plan for nutrition programming.” | Policy |
| India | “For understanding the coverage in different states and districts of the country. For sensitizing the programme managers about the coverage.” | Monitoring |
| India | “Reference for future studies and secondary data analysis.” | Research |
| India | “To understand compliance.” | Monitoring |
| India | “For prevalence of anaemia pre- and post-supplementation.” | Research |
| India | “Research and analysis.” | Research |
| India | “Programme planning/programme implementation/planning interventions.” | Implementation |
| Indonesia | “For capacity-building of public health human resources in field.” | Other |
| Kenya | “Improve decision-making on country-level policies and programme design, implementation and monitoring.” | Monitoring/implementation/  policy |
| Kenya | “Assessing progress, inform programming.” | Monitoring |
| Kenya | “Assess implementation gaps; develop campaign and educational priorities.” | Monitoring/policy |
| Malawi | “Interview.” | Other |
| México | “Magnitud y distribución de consumo, así como su posible contribución a la dieta de las mujeres y niños.” | Monitoring/research |
| Moldova | “To assess pr. efficiency, esp. on compliance (no. women who took iron pills > 90days).” | Monitoring |
| Nepal | “Try to identify group of mothers who have not consumed IFA. ” | Monitoring |
| Pakistan | “It is used to evaluate programme coverage and modification in the implementation strategies.” | Monitoring/implementation |
| Pakistan | “Cross-sectional, random sampling.” | Research |
| Peru | “Porcentaje que recibe suplementos. ” | Monitoring |
| Philippines | “Public health programme implementation of iron-folic acid for prenatal care and monitoring of micronutrient deficiencies among women and children.” | Monitoring/implementation |
| Sri Lanka | “Policy.” | Policy |
| Sri Lanka | “To identify the geographic locations to be focused to improve coverage and for advocacy to rectify the possible reasons for low coverage.” | Monitoring |
| Syria | “For upcoming nutrition strategy.” | Policy |
| Uruguay | “En realidad solamente los analiza y publica anualmente el Hospital que tiene mayor número de nacimientos en el país. ” | Other |
| Uruguay | “Por el mal planteamiento no puede ser empleadas para coberturas en menores de 5 años.” | Other |
| USA | “For developing research ideas, writing grants and preparing for class.” | Research |
| USA | “In countries, we usually find out whether mothers have received the tablets, and how many, so that we can see if there are gaps in what women are receiving during antenatal care.” | Monitoring |
| Venezuela | “Poco uso en cuanto al seguimiento de programas de salud pública en este tema.” | Monitoring |
| *Not mentioned* | “Depending on the coverage, and usage levels of IFA by the beneficiaries, we can provide our recommendations to act upon immediately to focus on the increase of awareness levels of beneficiaries, if stocks are not available, we recommend to ensure that the stocks should be available. Ultimately this data useful for the programme implementers and programme managers and health specialists.” | Implementation |

Note: verbatim responses edited slightly for clarity.

## Table A 15: Reported days of consumption by recall period among postpartum women who consumed iron tablets/syrup for at least one day in Afghanistan, Myanmar and Tanzania

| **Afghanistan** | | | | | | | | | |
| --- | --- | --- | --- | --- | --- | --- | --- | --- | --- |
| **Days** | Don’t know | | 1–29 | | 31–300^*^ | | 30, 60, 90, 120, 150, 180 | | Total |
|  | N | % | N | % | N | % | N | % | N |
| **Recall period** |  |  |  |  |  |  |  |  |  |
| < 1 year | 291 | 11.2 | 844 | 32.6 | 392 | 15.1 | 1062 | 41.0 | 2589 |
| 1 to < 2 years | 224 | 9.7 | 743 | 32.1 | 381 | 16.5 | 967 | 41.8 | 2315 |
| 2 to < 3 years | 155 | 9.6 | 625 | 38.9 | 239 | 14.9 | 588 | 36.6 | 1607 |
| 3 to < 4 years | 134 | 13.0 | 381 | 37.0 | 128 | 12.4 | 386 | 37.5 | 1029 |
| 4 to < 5 years | 51 | 9.2 | 216 | 39.0 | 70 | 12.6 | 217 | 39.2 | 554 |
| Total | 855 | 10.6 | 2809 | 34.7 | 1210 | 14.9 | 3220 | 39.8 | 8094 |
| **Myanmar** | | | | | | | | | |
| **Days** | Don’t know | | 1–29 | | 31–300^*^ | | 30, 60, 90, 120, 150, 180 | | Total |
|  | N | % | N | % | N | % | N | % | N |
| **Recall period** |  |  |  |  |  |  |  |  |  |
| < 1 year | 8 | 1.2 | 42 | 6.1 | 130 | 18.8 | 510 | 73.9 | 690 |
| 1 to < 2 years | 12 | 1.6 | 34 | 4.5 | 130 | 17.3 | 576 | 76.6 | 752 |
| 2 to < 3 years | 10 | 1.7 | 40 | 6.7 | 92 | 15.5 | 453 | 76.1 | 595 |
| 3 to < 4 years | 12 | 2.1 | 53 | 9.2 | 94 | 16.4 | 414 | 72.3 | 573 |
| 4 to < 5 years | 13 | 2.9 | 29 | 6.6 | 64 | 14.5 | 336 | 76.0 | 442 |
| Total | 55 | 1.8 | 198 | 6.5 | 510 | 16.7 | 2289 | 75.0 | 3052 |
| **Tanzania** | | | | | | | | | |
| **Days** | Don’t know | | 1 – 29 | | 31–300^*^ | | 30, 60, 90, 120, 150, 180 | | Total |
|  | N | % | N | % | N | % | N | % | N |
| **Recall period** |  |  |  |  |  |  |  |  |  |
| < 1 year | 22 | 1.3 | 293 | 17.6 | 133 | 8.0 | 1218 | 73.1 | 1666 |
| 1 to < 2 years | 20 | 1.2 | 351 | 21.4 | 127 | 7.7 | 1142 | 69.6 | 1640 |
| 2 to < 3 years | 30 | 2.9 | 233 | 22.8 | 74 | 7.2 | 684 | 67.0 | 1021 |
| 3 to < 4 years | 23 | 3.2 | 155 | 21.7 | 37 | 5.2 | 499 | 69.9 | 714 |
| 4 to < 5 years | 17 | 3.5 | 92 | 19.1 | 30 | 6.2 | 343 | 71.2 | 482 |
| Total | 112 | 2.0 | 1124 | 20.4 | 401 | 7.3 | 3886 | 70.4 | 5523 |
| ^*^Except 60, 90, 120, 150, 180 | | | | | | | | | |

## Table A 16: Evaluation of mean and standard error of consumption by recall period in DHS datasets

| **Recall period** | **Afghanistan** | | | **Colombia** | | | **Myanmar** | | | **Tanzania** | | |
| --- | --- | --- | --- | --- | --- | --- | --- | --- | --- | --- | --- | --- |
|  | Mean days | *SE* | *p* | Mean months | *SE* | *p* | Mean days |  | *p* | Mean days |  | *p* |
| Overall (0-5 years) | 46.2 | 2.02 | <0.001 | 5.4 | 0.04 | 0.0013 | 112.1 | 1.7 | 0.0050 | 55.8 | 0.88 | 0.0011 |
| < 1 year | 46.4 | 2.15 |  | 5.5 | 0.10 |  | 114.6 | 3.12 |  | 59.3 | 1.39 |  |
| 1 to <2 years | 50.7 | 4.23 |  | 5.4 | 0.09 |  | 117.2 | 2.68 |  | 55.4 | 1.54 |  |
| 2 to < 3years | 40.5 | 1.80 |  | 5.4 | 0.09 |  | 111.3 | 3.03 |  | 53.8 | 1.84 |  |
| 3 to <4 years | 46.1 | 4.68 |  | 5.3 | 0.12 |  | 105.2 | 3.24 |  | 52.8 | 1.73 |  |
| 4 to <5 years | 43.2 | 3.19 |  | 5.7 | 0.10 |  | 109.3 | 3.79 |  | 53.1 | 2.62 |  |
| SE: Standard error | | | | | | | | | | | | |
